# Functional characterization of three *Azotobacter chroococcum* alginate-modifying enzymes related to the *Azotobacter vinelandii* AlgE mannuronan C-5-epimerase family

**DOI:** 10.1038/s41598-020-68789-3

**Published:** 2020-07-27

**Authors:** Agnieszka Gawin, Lisa Tietze, Olav A. Aarstad, Finn L. Aachmann, Trygve Brautaset, Helga Ertesvåg

**Affiliations:** Department of Biotechnology and Food Science, NTNU Norwegian University of Science and Technology, Sem Sælandsvei 6/8, 7491 Trondheim, Norway

**Keywords:** Biochemistry, Biotechnology, Molecular biology

## Abstract

Bacterial alginate initially consists of 1–4-linked β-D-mannuronic acid residues (M) which can be later epimerized to α-*L*-guluronic acid (G). The family of AlgE mannuronan C-5-epimerases from *Azotobacter vinelandii* has been extensively studied, and three genes putatively encoding AlgE-type epimerases have recently been identified in the genome of *Azotobacter chroococcum*. The three *A. chroococcum* genes, here designated *AcalgE1*, *AcalgE2* and *AcalgE3*, were recombinantly expressed in *Escherichia coli* and the gene products were partially purified. The catalytic activities of the enzymes were stimulated by the addition of calcium ions in vitro. AcAlgE1 displayed epimerase activity and was able to introduce long G-blocks in the alginate substrate, preferentially by attacking M residues next to pre-existing G residues. AcAlgE2 and AcAlgE3 were found to display lyase activities with a substrate preference toward M-alginate. AcAlgE2 solely accepted M residues in the positions − 1 and + 2 relative to the cleavage site, while AcAlgE3 could accept either M or G residues in these two positions. Both AcAlgE2 and AcAlgE3 were bifunctional and could also catalyze epimerization of M to G. Together, we demonstrate that *A. chroococcum* encodes three different AlgE-like alginate-modifying enzymes and the biotechnological and biological impact of these findings are discussed.

## Introduction

Alginate is an industrially important linear copolymer consisting of 1–4-linked sugar residues of β-D*-*mannuronic acid (M) and its C-5 epimer, α-L-guluronic acid (G). It is the most abundant polysaccharide in brown seaweed, from which it is commercially extracted^1^, but it can also be produced by bacteria belonging to the genera *Azotobacter*^2^ and *Pseudomonas*^3^. The physicochemical properties of alginate depend on the relative amount and distribution of M and G residues arranged in M-, G- and MG-blocks^4,5^. As alginate initially is synthesized as mannuronan, G residues originate from mannuronan C-5-epimerases acting selectively at the polymer level, each introducing the specific epimerization pattern^6^. All studied alginate-producing bacteria encodes a periplasmic mannuronan C-5-epimerase that is a part of the alginate biosynthesis and export protein complex.

A family of seven calcium-dependent, secreted mannuronan C5 epimerases (AlgE1–AlgE7) has been identified in the soil bacterium *Azotobacter vinelandii*^7^, and the biochemical properties of these enzymes have been extensively characterized due to their potential biotechnological application^7–10^. The AlgE epimerases are modular enzymes composed of one or two catalytic A-modules (about 385 amino acids) and one to seven regulatory R-modules (about 153 amino acids)^7,11^. The enzymes are exported through a dedicated ABC-transporter complex^12^. Although the A-modules alone are sufficient for epimerization, the R-modules function as an additional source of calcium for the catalytic part and may stimulate binding of the epimerase to the substrate^10,13–15^. Despite structural homology within each group of their modules, different AlgE epimerases introduce either alternating M and G residues or continuous stretches of G residues of different lengths^11,16^.

*Azotobacter* sp. are known for their ability to enter a dormant life stage called cyst, in which the bacterium is enclosed in a capsule comprised of several biopolymers^17^. For *A. vinelandii*, it has been shown that alginates containing G-blocks are necessary for maintaining a viable cell within the cyst coat^18^. It has been suggested that the diverse enzymatic activities displayed by AlgE-type epimerases are required by *A*. *vinelandii* at different life stages and under varying environmental conditions^7^. The genome of another soil bacterium of the genus *Azotobacter*, *A*. *chroococcum* strain ATCC 4412, has been sequenced^19^, and found to encode three putative AlgE-like proteins^20^. The predicted *Achr_39550* gene product is homologous to the *A. vinelandii* mannuronan C-5-epimerase AlgE2. AlgE2 is composed of one A-module containing the catalytic site followed by four Ca^2+^-binding R-modules that participate in alginate binding^15^. AlgE2 can introduce single G residues and G residues next to pre-existing G-blocks, but not within alternating MG sequences, and the relative amount of introduced G-blocks increases with decreasing concentration of calcium^11,21^.

**Figure 1:**
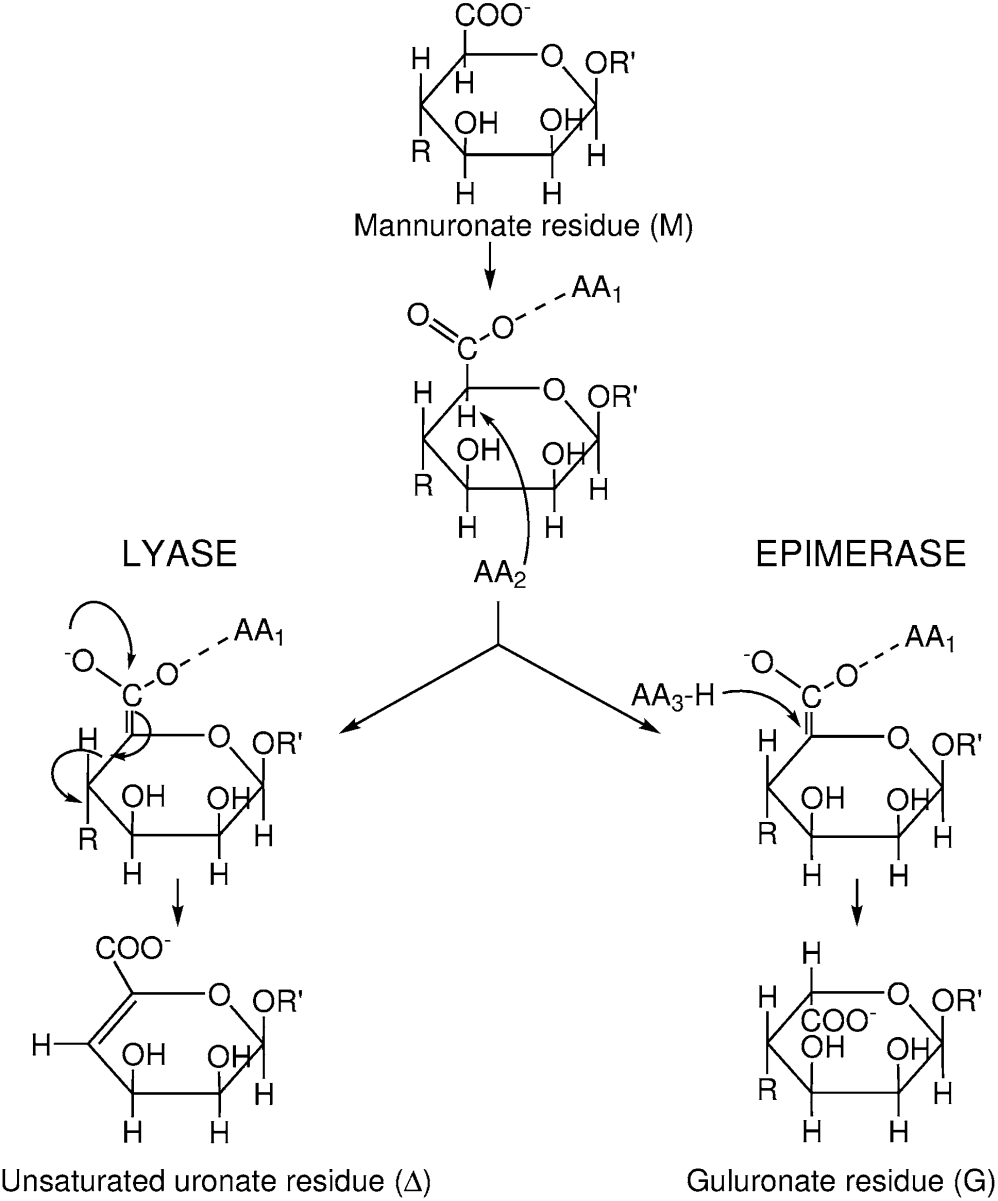
The reaction mechanisms of mannuronan C-5-epimerases and alginate lyases. The figure is based on Gacesa^22^. AA_#_ refers to catalytic active amino acid residues on the enzymes. AA_1_ neutralizes the carboxylate group and AA_2_ abstracts C5 proton. In the case of lyase reaction, a β-elimination, an electron transfer from the carboxyl group results in the formation of a double bond between C4 and C5 and cleavage of the glycosidic linkage. For the epimerase, AA_3_ donates a proton to the opposite face of the sugar ring. This results in a flip from ^4^C_1_ (M) to ^1^C_4_ (G) conformation. Chemical structures of mannuronate and guluronate residues were drawn using ChemBioDraw Ultra 14.0 (CambridgeSoft, Cambridge, MA, USA).

The *Achr_39560* and *Achr_39570* genes in *A. chroococcum* both encode proteins homologous to the extracellular bifunctional *A. vinelandii* mannuronan C-5-epimerase and alginate lyase AlgE7^20^. Lyase and epimerase reactions share the first steps (Fig. 1)^22^; the negative charge on the carboxylate anion is neutralized by an ionic bond with an amino acid residue (AA_1_) or Ca^2+^ followed by abstraction of the C5-proton by a second amino acid residue (AA_2_). Then an epimerase needs a third amino acid residue (AA_3_) to donate a proton to C5 from the opposite side of the sugar ring. In a lyase reaction, an electron transfer from the carboxyl group results in the formation of a double bond between C4 and C5 and a cleavage of the glycosidic linkage. This is often facilitated by an amino acid residue acting as a base^23^. Both reactions are catalyzed by the same single catalytic part (A-module) of AlgE7, which is succeeded by three R-modules^24^. The enzyme cleaves mainly G-MM and/or G-GM bonds in alginate, and less preferably M-MM and/or M-GM sequences. NMR spectroscopy has revealed that G residues dominate at the nonreducing ends of the degraded alginate even if poly-mannuronic acid is used as a substrate. It has, therefore, been suggested that AlgE7 introduces G-blocks, probably to facilitate the lyase pathway^25^.

In this study, we report the in vitro determined biochemical properties of the AlgE-type epimerases/lyases encoded by the *A. chroococcum* genes *Achr_39550, Achr_39560 and Achr_39570.* The corresponding proteins were produced recombinantly in *Escherichia coli,* to determine their substrate specificities, epimerization pattern, potential lyase activity and the composition of the epimerized/degraded alginate. The findings show that *A. chroococcum* encodes only one monofunctional secreted mannuronan C-5 epimerase, Achr_39550, and not six like *A. vinelandii* do. On the other hand, the bifunctional AlgE7-like enzymes Achr_39560 and Achr_39570, differ in their substrate specificities indicating that they may have different functions. Based on these results, the genes are hereafter designated *AcalgE1* (*Achr_39550*), *AcalgE2* (*Achr_39560*) and *AcalgE3* (*Achr_39570*) and the corresponding proteins denoted AcAlgE1, AcAlgE2 and AcAlgE3, respectively.

## Results

### Amino acid sequence analysis of the deduced AcAlgE1, AcAlgE2, and AcAlgE3 proteins from *A. chroococcum*

**Figure 2:**
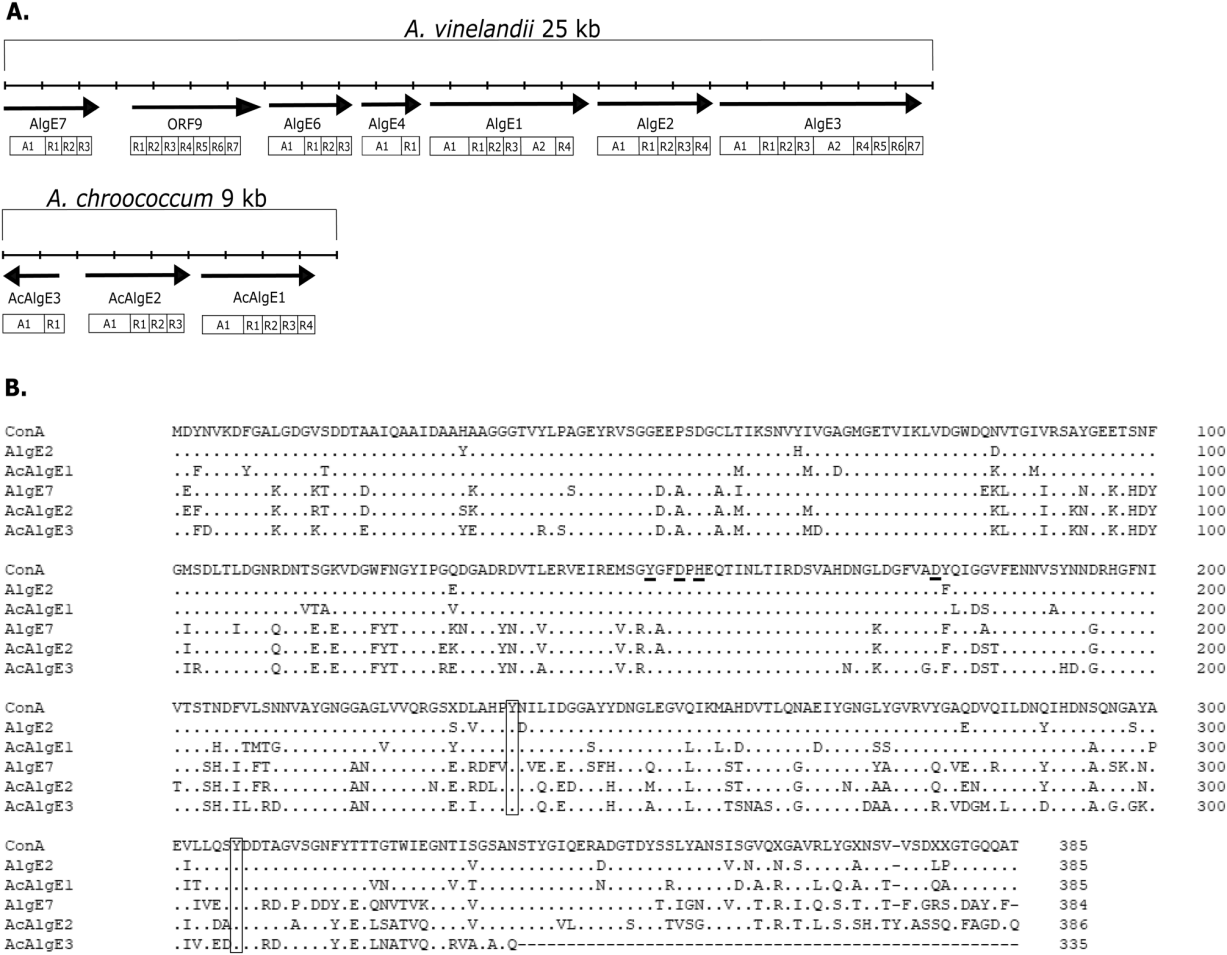
**(A)** The gene clusters encoding the AlgE epimerases in *A. vinelandii* and in *A. chroococcum.* The modular structures of each protein are depicted beneath the corresponding genes. **(B)** Alignment of the A modules of *A. vinelandii* AlgE2 and AlgE7 and *A. chroococcum* AcAlgE1, AcAlgE2, and AcAlgE3. All proteins are compared to ConA, a consensus sequence derived from the nine A-modules contained in *A. vinelandii* AlgE1-AlgE7. The active site residues are underlined in the ConA sequence. The conserved Tyr^235^ and Tyr^307^ are marked with brackets.

All but one of the seven *algE* genes of *A. vinelandii* are found in one gene cluster. The three genes encoding the AlgE-like enzymes of *A. chroococcum* are also clustered (Fig. 2A), and the clusters in both species have the same neighboring genes. As in the case of AlgE2, AcAlgE1 is composed of one A-module followed by four R-modules. The three R-modules of AcAlgE2 were found to be in the same position relative to the A-module as in AlgE7. In contrast to AcAlgE2, AcAlgE3 is missing 47 amino acids of the A-module and contains only one R-module, which is homologous to the R3 module of AlgE7. This might be a result of a gene duplication and reversion followed by an internal deletion.

Based on the amino acid sequences of the A-modules in AlgE1-7, we constructed a consensus sequence, denoted ConA. The sequences of the A-modules of AcAlgE1, AcAlgE2, and AcAlgE3 were aligned against ConA (Fig. 2B), and the percentage of identity for all of them was 60% or more. All three predicted *A. chroococcum* proteins contain the conserved Tyr^149^, Asp^152^, His^154^, and Asp^178^ amino acids residues of the catalytic site of mannuronan C-5-epimerases (Fig. 2B)^20^.

In an earlier study^26,27^, it was found that all *A. vinelandii* epimerases that are able to insert G next to preexisting G residues have conserved tyrosine residues at positions 235 and 307. Our analysis shows that the three *A. chroococcum* enzymes all contain these two tyrosine residues (Fig. 2B), and this could indicate that all three are able to form some GG sequences.

### Recombinant expression and purification of AcAlgE1, AcAlgE2, and AcAlgE3 proteins in *E. coli*

In order to investigate in vitro activities of the enzymes, DNA fragments corresponding to *AcAlgE1*, *AcAlgE2*, and *AcAlgE3* coding regions were cloned in the plasmid vector pVB1-251-bla_Kan under transcriptional control of the inducible *Pm* promoter and the resulting expression vectors, pAG550, pAG560, and pAG570, respectively, were transformed to *E. coli* RV308. The recombinant strains were cultivated in shake flasks and recombinant expression was induced before harvesting. The crude extracts were prepared, and the recombinant proteins were partially purified by ion-exchange chromatography (Supplementary Figure S1) (see “Materials and methods”). The purified proteins where then subjected to biochemical characterizations (see below).

### AcAlgE1 displays C-5 epimerase activity in vitro

**Figure 3:**
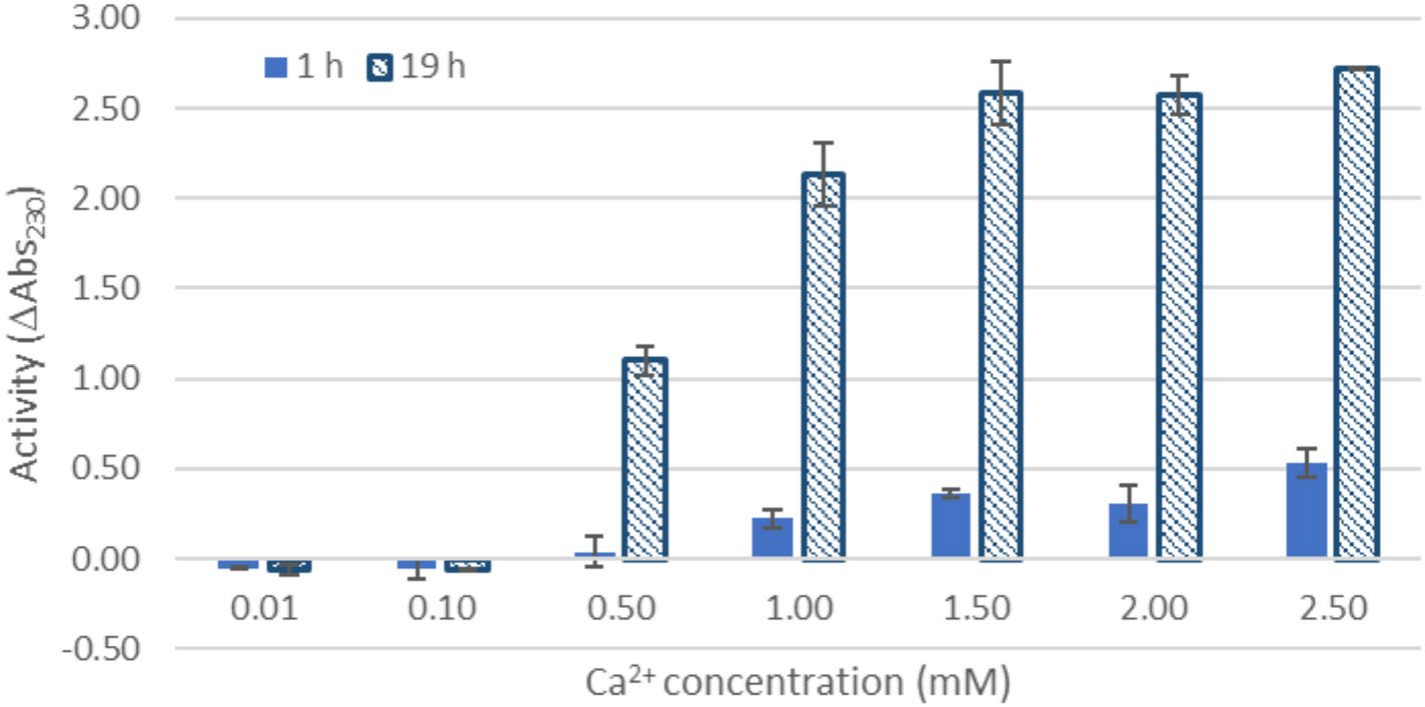
Epimerase activity of AcAlgE1 as a function of calcium. Mannuronan was epimerized for 1 h (filled) and 19 h (cross-hatched) with AcAlgE1 at different calcium concentrations before the G-lyase AlyA was added and the incubation continued for two hours. The activity is given as the increase in absorbance at 230 nm.

The purified AcAlgE1 protein was analyzed for epimerase activity using mannuronan (F_G_ = 0.0, F_G_ denotes the fraction of guluronic acid) as a substrate in the coupled enzyme assay as described in Materials and Methods. The monitored increase of A_230nm_ corresponded to the formation of unsaturated uronic acid residues resulting from degradation of the epimerized sample with AlyA that cleaves both G-M and G-G linkages, but not the M-M linkages found in mannuronan. Initial analysis revealed that AcAlgE1 displays epimerase activity. As expected, calcium ions were required by AcAlgE1 for epimerization, and a calcium concentration of at least 1.5 mM was needed for the optimal activity of the enzyme under the tested conditions (Fig. 3).

Next, AcAlgE1 was tested for lyase activity using LF 10/60 alginate as a substrate, which contains a mixture of all possible bonds found in alginates. No increase in A_230nm_ was detected when the substrate was treated with AcAlgE1 indicating that this protein has no lyase activity under the tested conditions (data not shown).

### Characterization of the epimerization pattern of AcAlgE1

A ^1^H-NMR analysis of mannuronan (F_G_ = 0.0) incubated with AcAlgE1 confirmed that this enzyme displays epimerase activity (Table 1). Since the initial changes in the epimerized substrate are known to affect the activity of epimerases, spectra representing different degrees of epimerization at three different time points (12, 24, and 48 h) were recorded. The data showed that AcAlgE1 introduces mainly G-blocks, while the amount of alternating structures decreases during the reaction. The high degree of epimerization after 48 h and an average G-block length well above 10 indicate that mannuronan is converted into a polymer with a predicted capacity to form strong gels with calcium^8,28^.

**Table 1:** Composition, sequence parameters, and M_w_ for mannuronan (Mw = 140 kDa) incubated with AcAlgE1 for 12–48 h.

Time (h)	FG	F_M_	FGG	FMG/GM	FMM	FMGG/GGM	FMGM	FGGG	NG > 1	M_w_ (Da)
12	0.586	0.414	0.508	0.078	0.335	0.041	0.039	0.466	13.2	1.1 × 10^5^
24	0.831	0.169	0.762	0.068	0.101	0.045	0.028	0.718	17.9	1.1 × 10^5^
48	0.865	0.135	0.802	0.063	0.072	0.043	0.023	0.760	19.6	1.1 × 10^5^

F_G_ denotes the fraction of guluronic acid. The fractions of different diads and triads are indicated with two and three letters, respectively. N_G>1_ is the average G-block length calculated as (F_G_ − F_MGM_)/F_MGG_.

**Figure 4:**
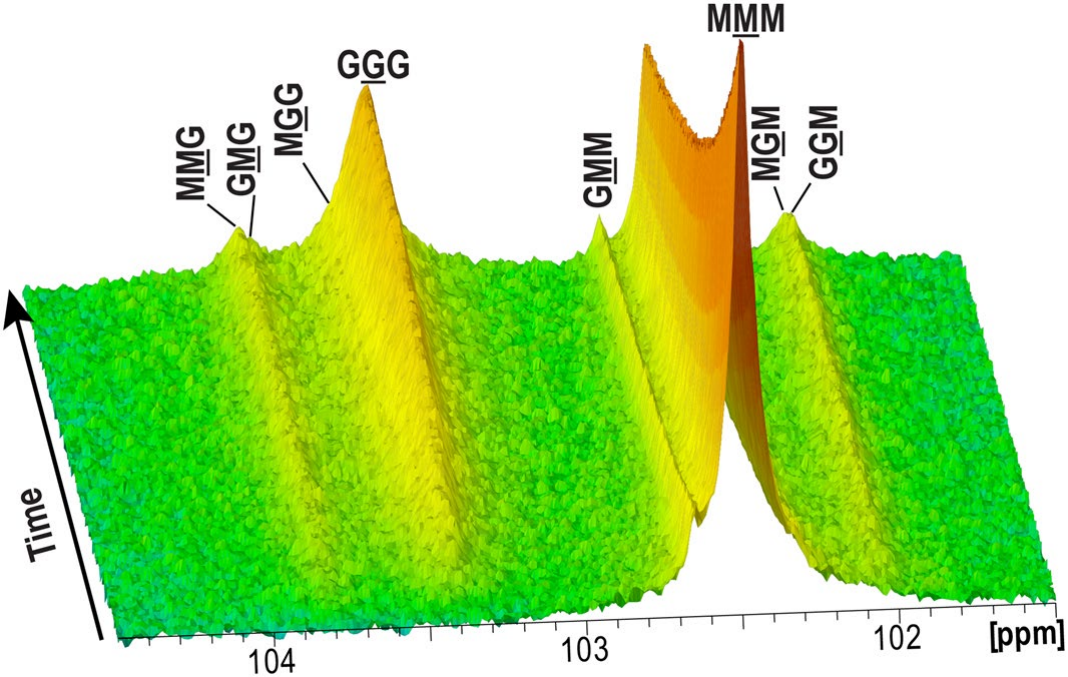
Time-resolved NMR showing epimerization of ^13^C-labeled mannuronan with AcAlgE1. The position of the triads in the spectra is indicated, and the M or G moiety generating the signal is underlined. The spectra were recorded, processed and analyzed using TopSpin 3.6pl7 software (Bruker BioSpin) and finalized in Adobe Illustrator 2020 (Adobe Systems, USA).

In order to better understand the mode of action of AcAlgE1, the progress of epimerization was monitored by time-resolved NMR using ^13^C-labeled mannuronan as a substrate. As seen in Fig. 4, the change in block composition in the substrate was observed as the relative rate by which the signal for monomer triads (MMM, GGG, GMG, and MGM) develop. AcAlgE1 displays an almost immediate formation of G-blocks (seen as an increase in peak marked GGG). This is accompanied by a simultaneous decline in the content of M-blocks (MMM) as well as a slow accumulation of alternating MG-blocks (GMG and MGM). The enzyme seems to prefer to introduce G residues next to a preexisting G residue resulting in G-block formation detected as a slow increase in MGG and GGM levels indicating the start and end of the G-blocks, respectively. Moreover, MMG and GMM peaks seem to slowly increase during the reaction indicating the formation of new G-blocks or single G residues. Overall, the results suggest that AcAlgE1 makes G-rich alginate with a minor fraction of alternating MG-block structure. Therefore, the enzyme displays similar functional properties as AlgE2 and AlgE6^7,20^.

**Figure 5:**
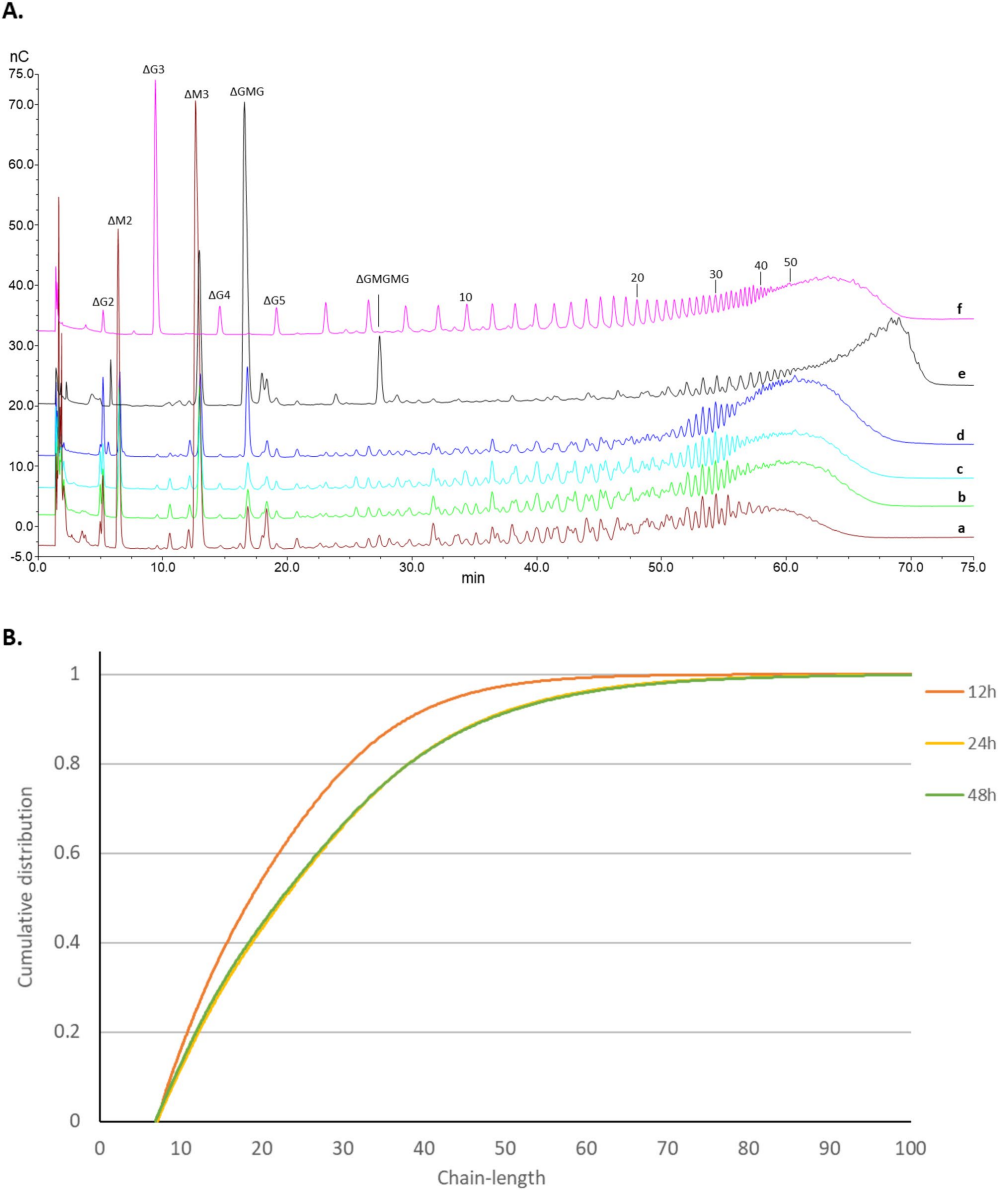
Distribution of G-blocks in mannuronan epimerized with AcAlgE1. The samples were degraded by the M lyase from *H. tuberculata*. A: Overlaid HPAEC-PAD chromatograms of mannuronan incubated with AcAlgE1 for **(a)** 12,** (b)** 24, and **(c)** 48 h. Mannuronan epimerized with **(d)** AlgE6 (F_G_ = 0.88) and **(e)** AlgE1 (F_G_ = 0.80) are included for comparison and **(f)** G-block (F_G_ > 0.97) partially degraded by G-lyase from *K. pneumoniae* is shown as a standard. **(B)** Cumulative number of fractions as a function of G-block length of mannuronan epimerized with AcAlgE1 for 12, 24, and 48 h calculated from SEC-MALS analysis. The oligomers with chain length less than 7 were not included since these mainly contain degradation products of M and MG sequences.

While the NMR data showed relative amounts of different monomers, dimers, and trimers in the oligomer, HPAEC-PAD and SEC-MALS were used to analyze the distribution of different oligomers in the epimerized substrate. For this purpose, the samples of mannuronan incubated with AcAlgE1 for 12, 24, and 48 h were degraded with an M-M/G-M specific lyase from *Haliotis tuberculata*. As the distribution of G-block lengths introduced by the *A. vinelandii* epimerases AlgE1 and AlgE6 has been studied previously^8,29^, the chromatograms of their products degraded with the M-M/G-M lyse were used for comparison. All HPAEC-PAD chromatograms are given in Fig. 5A. In this case, the method does not allow for the quantification of each oligomer due to a lack of resolution for the longer G-blocks (DP > 20). This is probably caused by the heterogeneity of the ends of G-blocks where the M-lyase is unable to cleave remaining M residues (forming e.g. MGG sequence) and can be seen by a comparison with pure G oligomers (G-blocks partially degraded by G-lyase from *Klebsiella pneumoniae*). In fact, the previous study estimated the actual G-block length to be 1–3 units shorter than the oligomers^30^. The chromatogram profiles clearly depict that the distribution of G-blocks found in mannuronan epimerized with AcAlgE1 is more similar to the distribution pattern obtained with AlgE6 than with AlgE1. Most of the G-blocks formed by AcAlgE1 and AlgE6 have DP between 20 and 50, while AlgE1 introduces longer G-blocks. In addition, the amount of MG sequences is lower in the AcAlgE1epimerized alginate than in the product of AlgE1 as seen as a difference in the content of MG tetramers (ΔGMG) and hexamers (ΔGMGMG). The SEC-MALS analysis (Fig. 5B) showed that the block length increases slightly with the increasing G content from 59% (12 h) to more than 80% (24 and 48 h). No significant difference in the chain-length distribution between the samples containing 83% (24 h) and 87% (48 h) of the G residues can be observed indicating that condensation of G-blocks with a few M units in between is only rarely catalyzed by AcAlgE1. Although no increase in A_230nm_ during the lyase activity assay was detected for AcAlgE1, a slight decrease in M_w_ from the initial 140 kDa to 110 kDa was measured by SEC-MALS after 12 h incubation with AcAlgE1, no further decrease was observed in the samples incubated for a longer time.

### AcAlgE2 and AcAlgE3 both display lyase activities in vitro

**Figure 6:**
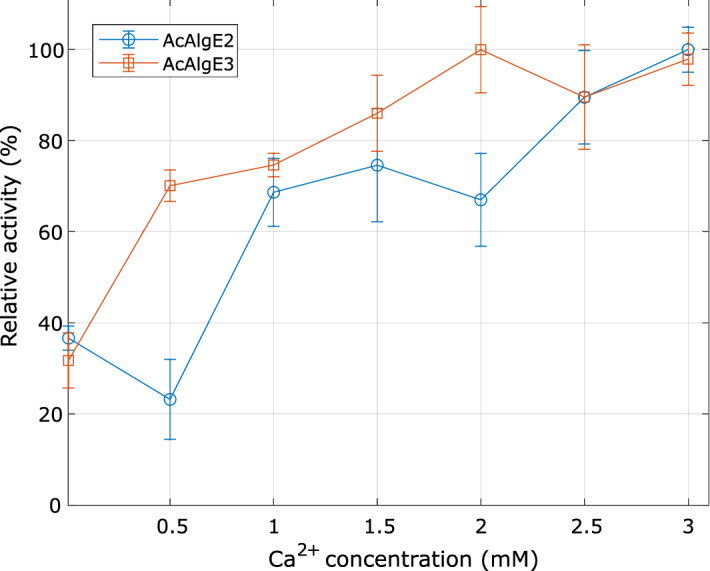
Lyase activity of AcAlgE2 and AcAlgE3 as a function of the calcium concentration. The highest activity (U/min) within the studied range of calcium concentrations was set as 100% for each enzyme. LF 10/60 alginate (1 mg/mL) was used as a substrate.

As AcAlgE2 and AcAlgE3 show sequence homology to the bifunctional *A. vinelandii* AlgE7, we suspected that these enzymes might display lyase activities. Accordingly, partially purified proteins were tested for lyase activity using LF 10/60 alginate as a substrate. For both enzymes, a significant increase in A_230_ was detected, even though AcAlgE3 encodes an enzyme with a shorter version of the catalytic A-module. The activities of AcAlgE2 and AcAlgE3 were also affected by the addition of calcium in the reaction mixture (Fig. 6).

Alginate consists of two different monomers and may contain up to four different bonds between these residues, and many lyases cleave only some of these bonds. In order to investigate the catalytic activities of AcAlgE2 and AcAlgE3 on different bonds, three structurally different alginates were tested; poly-M (F_G_ = 0.05) which contains almost only M–M bonds, poly-G (F_G_ = 0.95) which predominantly contains G-G bonds and MG-alginate (F_G_ = 0.46) which mostly contains M–G and G–M bonds. For both enzymes, poly-M appeared to be the best substrate, while poly-G was not utilized as a substrate by any of the enzymes. Only AcAlgE3 utilized MG-alginate as a substrate. However, due to the presence of 8% M–M bonds in the poly-MG-alginate, the difference in activity on the different substrates cannot be quantified.

### Time-resolved ^13^C NMR spectroscopy analysis of the reaction products confirms that AcAlgE2 and AcAlgE3 are different bifunctional enzymes with lyase and epimerase activities

**Figure 7:**
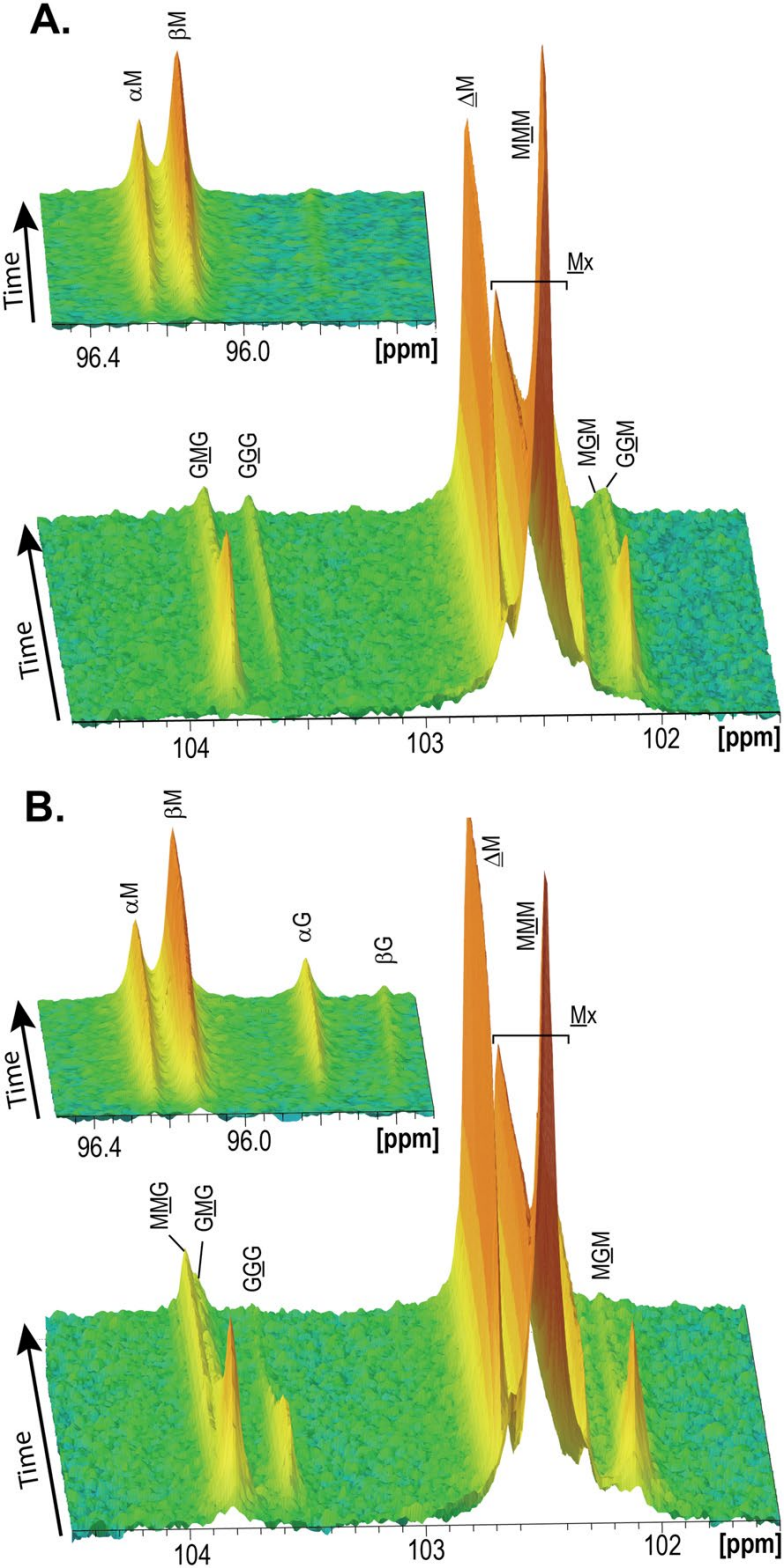
Time resolved NMR spectra of ^13^C-labeled mannuronan treated with **(A)** AcAlgE2 and **(B)** AcAlgE3. The position of the triads in the spectra is indicated, and the M or G moiety generating the signal is underlined. Signals marked with symbols indicate the unsaturated end residues from the β-elimination (Δ = 4-deoxy-l-erythro-hex-4-enepyranosyluronate), and the α- or β-reducing ends. The spectra were recorded, processed and analyzed using TopSpin 3.6pl7 software (Bruker BioSpin) and finalized in Adobe Illustrator 2020 (Adobe Systems, USA).

Since the epimerization assay is a coupled assay in which alginate lyases are used to measure epimerization, the method is not suitable for testing any epimerase activity of potentially bifunctional enzymes. Therefore, time-resolved NMR spectra were recorded to provide detailed information on the mode of action of AcAlgE2 and AcAlgE3 (Fig. 7). ^13^C-labeled mannuronan was used as a substrate for the analysis ensuring that any G signal would be caused by epimerase activity. A rapid decrease in the content of M blocks (MMM) and an increasing signal from unsaturated residues next to an M residue (ΔM) were observed for both enzymes confirming their lyase activities. Moreover, MGM and GMG rapidly appeared, showing epimerase activity for both enzymes. The subsequent decrease in these signals could result from a combination of either further epimerization into GGG or lyase degradation. Both AcAlgE2 and AcAlgE3 are able to form G-blocks (seen as GGG signal), while only AcAlgE3 seems to accept G-alginate as a substrate for degradation (seen as a later decrease of GGG signal and formation of the reducing end on G). For AcAlgE2 the GGM signal which signifies the number of G-blocks is increasing with time. Moreover, the spectra reveal a difference between the lyase activities of the two enzymes; while AcAlgE2 almost exclusively creates molecules with M at the reducing end resulting from cleavage of M–M and possibly M–G bonds, AcAlgE3 cleaves the chain after both M and G residues.

### Analysis of mannuronan degraded with AcAlgE2 and AcAlgE3 further discriminate their different lyase activities

**Figure 8:**
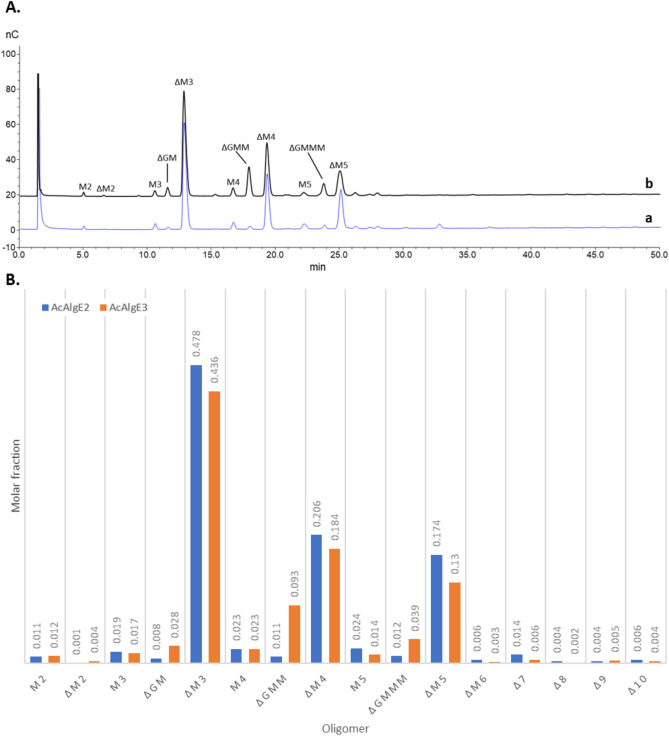
Fractions of different oligomers produced by AcAlgE2 and AcAlgE3. **(A)** HPAEC-PAD analysis of the NMR samples shown in Fig. 7 diluted to 0.1 mg/mL; poly-M degraded with **(a)** AcAlgE2, and **(b)** AcAlgE3. The following standards were used for peak identification (data not shown): mannuronan and poly-MG (F_G_ = 0.46) partially degraded by the M lyase from *H. tuberculata*, and mannuronan partially degraded by AlgL. **(B)** Molar fractions of the products from mannuronan incubated with AcAlgE2 and AcAlgE3. The quantification was done with response factors previously determined^36^ for M oligomers using a logarithmic fit: Rf_rel_ = 1.276 × ln (DP) + 0.3685, where DP is a degree of polymerization. Δ7–10 are unidentified unsaturated oligomers with at least one G unit. G = GulA, M = ManA, Δ, and the number denotes chain length, including Δ.

The AcAlgE2 and AcAlgE3 degradation products of mannuronan used for NMR spectroscopy were further analyzed by HPAEC-PAD. This allowed us to determine the amount of each fragment in the degradation mixture and identify the shortest oligomer that is accepted as a substrate by the lyases. In addition, since dimers and trimers containing G residues elute slightly different from those containing only M residues, the chromatograms would be a second way of showing if AcAlgE2 and AcAlgE3 were only lyases or, like AlgE7, could epimerize M to G. The results (Fig. 8) revealed that both enzymes produced mainly ΔM-containing trimers, tetramers, and pentamers. The product of AcAlgE3 contains also ΔGM and ΔGMM showing that the enzyme creates both ΔM and ΔG, while AcAlgE2 produces no or very low amounts of ΔG. Both enzymes seem to use hexamers as substrates, while pentamers are barely cleaved. Moreover, given the lower amount of dimers compared to trimers, the enzymes seem to prefer to cleave at least three residues away from both the reducing and nonreducing ends.

## Discussion

Alginate is currently manufactured from brown algae. However, much of this alginate has a G-content below 50%, while alginates with a higher G-content are needed for some medical and pharmaceutical applications^31,32^. The limited supply of alginates with a high content of G-blocks is currently limiting the development and use of such materials. Since in vitro epimerization can be used to increase the G-content and therefore the value of alginates with lower G-content, mannuronan C5 epimerases that catalyze this reaction have an industrial application. Studies have been made on both the natural *A. vinelandii* enzymes and hybrid epimerases in order to obtain alginate-modifying enzymes with novel properties and also to better understand the structure–function relationships in order to enable future tailoring of industrial epimerases^10,15,26,33^.

Different mannuronan C-5-epimerases have been shown to function according to two distinct mechanisms of alginate epimerization. The first of them is a processive mechanism that involves a random attack followed by sliding of the enzyme along the polymer chain during the epimerization process without dissociation of the enzyme–substrate complex. The other mechanism is a preferential attack in which the enzyme dissociates from the substrate after epimerization and binds to preferred M residues in consecutive attacks^9,34,35^. Determination of the epimerization pattern introduced by the mannuronan C-5-epimerase AcAlgE1 revealed that its affinity for the substrate depends on pre-existing G residues. This indicates that AcAlgE1, like AlgE2, might act by the preferred attack mechanism^26,34^. It was shown that AlgE2 is able to produce alginate with a relatively high content of G residues (up to 70%)^7,21^. However, alginate epimerized by AcAlgE1 reached an even higher degree of epimerization (up to 87%) and contained longer G-blocks. In fact, the G content and G-block distribution in the AcAlgE1-epimerized sample were more similar to the ones found in the product of AlgE6^8,29^. The long G-blocks generated by AlgE6 were shown to be associated with the formation of strong gels that shrink upon saturation with Ca^2+^ ions^8^ and the enzyme has been used to prolong G-blocks in natural and synthetic G-alginates^37^. Thus, the potential of AcAlgE1 for upgrading algal alginates for high-cost medical and pharmaceutical applications should be further explored.

The two bifunctional epimerases/lyases AcAlgE2 and AcAlgE3 studied here were shown to differ in terms of their enzymatic activities. AcAlgE2 activity resulted in the products M_red_ and ΔM, and eventually led to the accumulation of some G-blocks. AcAlgE3 was able to create all four possible products: M_red_, G_red_, ΔM and ΔG. Thus, both enzymes can be characterized as AlgE7-like due to their bifunctional epimerase/lyase activity. So far not much is known about which residues are important for the epimerase versus the lyase activities of AlgE7. Figure 1 indicates that difficulties in adding the proton from the other side if any of the neighboring residues are G residues, could result in an aborted epimerization. If the common step is most energy-demanding and the second step of the lyase reaction can take place spontaneously, this would explain the data. Alternatively, one of the amino acids specific for AlgE7 serves as the acid that has been suggested to aid in the last step of the lyase reaction. Figure 2B shows that the three bifunctional enzymes share many amino acid substitutions relative to the consensus sequence, and together with homology modeling, these might be used as starting points for site-directed mutagenesis in order to further elucidate the lyase mechanism of these enzymes.

The reason why *A. chroococcum* possesses two bifunctional AlgE7-like enzymes remains unknown. However, *A. vinelandii* encodes two secreted alginate lyases, AlgE7 and AlyA3^20^. Bacteria belonging to the genus *Azotobacter* are known for their ability to form a resting stage called cysts. It has been demonstrated that AlyA3 is necessary for cyst germination in *A. vinelandii,* and it was suggested that AlgE7 participates in the release of alginate associated with the cell surface^38^. AlgE7 does not make ΔG and has a similar size as AcAlgE2 (90 kDa), while AlyA3 can make all four products and has a similar size as AcAlgE3 (49 and 51 kDa, respectively). In the case of epimerases, it was suggested that a smaller size might facilitate binding to the substrate when it becomes less accessible for example as a result of oxidation^10^. The *A. chroococcum* gene does not encode an AlyA3 homologue, and given the similarities in their properties, AcAlgE3 might be needed to release the germinating cell from the *A. chroococcum* cyst coat.

Since several species of the genus *Azotobacter* have good plant growth promoting properties like nitrogen fixation and secretion of urea or amino acids, the ability to dissolve phosphorus from rock particles, synthesis of water-binding polysaccharides and anti-fungal compounds, they might be used to improve crop production. Indeed, *Azotobacter* sp. are already sold as a biofertilizer. Cysts can survive years of desiccation, and they could thus be a convenient way to spread these bacteria e.g. as part of the sowing process. For *A. vinelandii*, it has been shown that epimerized alginate is an important and necessary part of the cyst coat^17,39^. The structure of the *A. chroococcum* cyst is similar to that of *A. vinelandii*^40^, however, to our knowledge no studies on the chemical composition of the *A. chroococcum* cyst has been published.

The only study analyzing the G-content in alginates produced by *A. chroococcum* found that alginates from a late exponential phase culture contained only 10% of G residues^41^. This is lower than the numbers reported for wild type *A. vinelandii*, but similar to that of an *A. vinelandii* mutant expressing AlgE5 only^39,42^. It has been suggested that *A. vinelandii* encodes six different secreted epimerases because it needs different alginates at different life stages. It has also been observed that different parts of the cyst coat contain alginates with different G-content^17^. The structure of *A. vinelandii* alginate is known to depend on the culture conditions^43,44^, and the increasing environmental oxygen concertation leads to the formation of the polymer with higher molecular weight and greater content of G residues^45^. According to Sabra et al.^45^, these characteristics of the alginate play a decisive role in the protection of nitrogenase against oxygen. In turn, *A. chroococcum* has been reported to produce melanin from catechol and employ melanization as the mechanism protecting nitrogenase activity^46–48^. Still, in order to better understand the biological difference between the two species, studies on the composition of alginates produced by *A. chroococcum* and *A. vinelandii* at different life stages and in media with and without fixed nitrogen are needed.

The characterization of activities of the three *A. chroococcum* AlgE like enzymes has resulted in the identification of a new epimerase with potential industrial applications. Moreover, our results should contribute to a better understanding of the relationship between the primary structure and function of alginate-modifying enzymes.

## Methods

### Bacterial strains and growth conditions

*E. coli* DH5α (Bethesda Research Laboratories) was used for cloning purposes. *E. coli* RV308 (ATCC 31608) served as an expression host for production of the three enzymes. *A. chroococcum* strain B (ATCC 4412/NCIMB 8003) was used to clone the genes. *E. coli* was routinely grown at 37 °C with 225 rpm shaking in liquid LB medium (10 g/L tryptone, 5 g/L yeast extract, and 5 g/L NaCl), unless stated otherwise. *A. chroococcum* was cultivated in Burks medium^49^ at 30 °C with 225 rpm shaking. Solid media were prepared by adding 15 g/L agar to the media. For plasmid selection in *E. coli*, kanamycin was used at concentration 50 μg/mL. For protein expression, recombinant cells were grown in 3 × LB (500 mL) supplied with 5 mM CaCl_2_ in 3 L baffled shake flasks at 30 °C until reaching OD_600nm_ of 0.8–1.2 before induction with 1 mM m-toluic acid. Growth was continued overnight (~ 18 h) at 16 °C before harvesting the cells by centrifugation.

### Isolation of DNA from *A. chroococcum* and plasmid construction

Standard recombinant DNA procedures were performed as described by Sambrook and Russell^50^. Genomic DNA from *A. chroococcum* was isolated by using MasterPure Complete DNA and RNA Purification Kit (Epicentre). PCR was performed using the Q5 polymerase (New England Biolabs Inc.). Plasmids were purified with ZR Plasmid Miniprep kit (Zymo Research). Transformations were performed according to RbCl transformation protocol (New England Biolabs Inc.).

The Gibson assembly protocol^51^ was used for cloning the DNA sequences corresponding to *AcalgE1*, *AcalgE2*, and *AcalgE3* into pVB1-251 (Vectron Biosolutions AS, Trondheim). This vector is a derivative of pJBphOx-251^52^ and has a medium plasmid copy number and the *hok-sok* system for plasmid stability. The genes were placed under control of the inducible *Pm* promoter generating plasmids pAG550, pAG560 and pAG570, respectively.

### Enzyme purification

For preparation of AcAlgE2 and AcAlgE3 extracts, the cells were sonicated in 50 mL MC buffer [20 mM 3-(N-Morpholino)-propanesulfonic acid (MOPS), pH 6.9, 1 mM CaCl_2_] and centrifuged at 27,000 × g for 30 min. The supernatant was filtered through a membrane with a pore size of 0.22 μm and applied on a 5 mL anion exchange chromatography column (HiTrap Q HP, GE Healthcare). The enzymes were purified in Fast Protein Liquid Chromatography system (ÄKTA FPLC system, GE Healthcare) by using a continuous NaCl gradient (0 to 1 M) of 50 mM MOPS, pH 6.9, 1 mM CaCl_2_. Fractions were analyzed for epimerase and lyase activity, and the enzyme-containing fractions collected at approximately 0.6 M NaCl were used for further analysis. The total protein content was determined with NanoDrop One Microvolume UV–Vis Spectrophotometer (Thermo Scientific, DE, USA) and SDS-PAGE. AcAlgE1 was prepared as above, but 5 mM CaCl_2_ was used in all buffers, and the enzyme was further purified and concentrated using a 1 ml anion exchange chromatography column (HiTrap Q HP, GE Healthcare).

### Alginates used in this study

Protanal LF 10/60 TM sodium alginate (F_G_ = 0.67) from *Laminaria hyperborea* used for lyase activity assay was obtained from FMC Biopoly-Mer. For some substrate specificity analysis, poly-M (F_G_ = 0.05, M_w_ = 147 kDa) previously isolated from *Pseudomonas aeruginosa*^53^ was used as a substrate. PolyMG (F_G_ = 0.46 and F_GG_ = 0.00) was prepared by epimerization of M-alginate with recombinant mannuronan C-5-epimerase AlgE4^11^. The G-blocks (G-alginate, F_G_ = 0.95) were prepared from *L*. *hyperborea* as described by Haug et al.^54^. High molecular weight mannuronan (F_G_ = 0.0, M_w_ = 140 kDa) was produced from an epimerase negative AlgG mutant of *Pseudomonas fluorescens*^55^. ^13^C-1 enriched mannuronan (M_w_ = 106 kDa) was produced by growing *P. fluorescens* on a minimal media with 99% D-^13^C-1 fructose used as a carbon source^9^.

### Measurements of enzyme activities

Cleavage of the alginate by alginate lyase generates a double bond that absorbs at 230 nm, and lyase activity was measured as the increase in absorbance at 230 nm, as described earlier^52^, using 50 μl alginate (1 mg/mL), 50 μl buffer (20 mM MOPS, pH 6.9, 40 mM NaCl, 3 mM CaCl_2_, except for when the dependence on calcium ions were tested) and 5 μl enzyme. For the initial studies, a mixture of alginate (Protanal LF 10/60 TM) was used, while for substrate specificity analysis, polyM, poly-G, and poly-MG were used. The absorbance was measured every 10 s for 7.5 min with a SpectraMax Plus 384 Absorbance Microplate Reader (Molecular Devices, Wokingham, UK).

Epimerase activity was measured in a coupled assay described by Stanisci et al.^10^ using mannuronan (F_G_ = 0.0, 1 mg/mL) as substrate and 20 mM MOPS, pH 6.9, 40 mM NaCl, 2 mM CaCl_2_ as buffer, except for when the dependence on calcium ions were tested. Epimerization of M residues to G residues creates cleavable bonds for the alginate lyase AlyA from *K pneumoniae*, which only cleaves at G residues. In this way, epimerization can be assayed as the increase in absorbance at 230 nm after incubation with the lyase. This coupled assay is only semi-quantitative since the epimerase reaction is not stopped before the lyase is added, but it does give relative values.

### Endpoint and time-resolved NMR analysis of the alginate enzymatic products

These analyses were performed similarly to what has been reported earlier^9,27^. All alginate samples epimerized with AcAlgE1, AcAlgE2 and AcAlgE3 for the endpoint reaction were subjected to a two-step weak acid hydrolysis before the samples were analyzed by proton NMR^56^. As a chemical shift reference 5µL of an 1% TSP (3-(Trimethylsilyl)-propionic-2,2,3,3-d4 acid sodium salt) (Sigma-Aldrich, St. Louis, MO, USA) in 99.9% D_2_O was added. The residual calcium in the samples were removed with 20 µL of the 0.3 M chelator TTHA (triethylenetetramine-N,N,N′,N′′,N′′′,N′′′-hexaacetic acid) (Sigma-Aldrich, St. Louis, MO, USA). ^1^H-NMR spectra for endpoint reaction were recorded on BRUKER Avance IIIHD 400 MHz equipped with a 5 mm SmartProbe at 83 °C. The time-resolved spectra ^13^C-NMR spectra were recorded on BRUKER Avance IIIHD 800 MHz equipped with a 5 mm TCI cryoprobe at 25 °C. A stock solution of 10 mg/mL ^13^C-1 enriched poly-M (average DPn ~ 70) in 5 mM MOPS, pH 6.9 with 75 mM NaCl and 2 mM CaCl_2_ in 99.9% D_2_O was prepared for the time-resolved study of AcAlgE1, AcAlgE2 and AcAlgE3. Purified enzymes from anion exchange chromatography were subjected to buffer exchange using 5 mM MOPS, pH 6.9 with 75 mM NaCl and 27.5 mM CaCl_2_ in 99.9% D_2_O and concentrated to a final concentration of around 1.3–5 mg/mL using a spin column [Amicon Ultra, 0.5 mL, Ultracel membrane low binding regenerated cellulose (Merck Millipore, MA, USA)] with MWCO 3 kDa. Enzyme concentrations were determined with NanoDrop One Microvolume UV–Vis Spectrophotometer (Thermo Scientific, DE, USA) using ɛ 10,000 M/cm. 150 μL of ^13^C-1-enriched poly-M stock solution in a 3 mm NMR tube (LabScape Stream, Bruker LabScape Store) was preheated in the NMR instrument and 1D proton and carbon spectra were recorded before the time-resolved NMR in order to check that the sample has not been depolymerized or contaminated. 15 μL of the enzyme solution was added to the NMR tube with preheated substrate using a thin glass pipette and flipped at least three time to mix the sample. The sample was inserted into the preheated NMR instrument and the time-resolved experiment was initiated. The time-resolved experiment was recorded as a pseudo-2D spectrum by collecting a 1D carbon NMR experiment every 5 min totally 200–256 times. The 1D carbon spectrum used in the time-resolved (inverse gated proton decoupling) contains 8,192 data points and has a spectral width of 80 ppm, 48 scans with a flip angle of 30°, 2.1 s relaxation delay and total experiment time of 121 s. The spectra were recorded, processed and analyzed using TopSpin 3.6pl7 software (Bruker BioSpin).

### HPAEC-PAD

The NMR samples of mannuronan incubated with AcAlgE2 and AcAlgE3 were diluted to 0.1 mg/mL prior to analysis. The samples of alginate epimerized with AcAlgE1 from 12 to 48 h were degraded by M-lyase from *H. tuberculata* as previously described^30^.

Briefly, the samples (7.1 mg/mL) were dissolved in 200 mM NH_4_Ac, pH 7.2, 50 mM NaCl. M-lyase (0.02 U/mg substrate) was added and the reactions were incubated for 24 h at 30 °C. The samples were diluted to 1 mg/mL prior to analysis.

The following standards were made for peak identification as previously reported^29,30^: M-lysates of poly-M (F_G_ = 0.0), poly-MG (F_G_ = 0.46), poly-M epimerized with AlgE6 (F_G_ = 0.88) and AlgE1 (F_G_ = 0.80), partially acid hydrolysed poly-M and G-block (F_G_ > 0.97) degraded by G-lyase from *K. pneumoniae*.

High Performance Anion Exchange Chromatography (HPAEC) with Pulsed Amperometric Detection (PAD) were performed on a ICS 5000 + system (Thermo Scientific, DE, USA). Alginate samples (25 µL) were separated at 24 °C on a 4 × 250 mm IonPac AS4A Column with a 4 × 50 mm AG4A Guard Column at a flow rate of 1 mL/min. Samples were eluted with 100 mM NaOH and a linear sodium acetate gradient from 10 to 700 mM in 80 min using the gold Ag/AgCl carboquad waveform for detection. Data were processed using Chromeleon 7.1 software (Thermofisher, Sunnyvale, CA).

### Molecular weight measurements

Size-exclusion chromatography (SEC) with multi-angle laser light scattering (MALS) was performed at ambient temperature on an HPLC system consisting of a solvent reservoir, on-line degasser, HPLC isocratic pump, and automatic sample injector. Poly-M and epimerized samples (1 mg/mL) were analyzed on a TSKgel PW6000 column (Tosoh Bioscience, CA, USA) while the M-lyase degraded samples (7.1 mg/mL) were analyzed on serially connected TSKgel PWXL 2500 and 4000 columns (Tosoh Bioscience, CA, USA). The column outlet was connected to a Dawn HELEOS-II multi-angle laser light scattering photometer (Wyatt Technology, CA, USA)(λ0 = 663.8 nm) followed by Shodex RI-501 refractive index detector (Shodex, Tokyo, Japan). The eluent was 0.15 M NaNO_3_, 0.01 M EDTA (pH 6.0) and the flow rate was 0.5 mL/min. Samples were filtered (pore size 0.45 μm) before injection and each sample was analyzed twice with injection volume 50 and 100 μL. Data were collected and processed (with dn/dc = 0.150 mL/g) using the Astra (v. 7.3.0) software (Wyatt Technology, CA, USA).

## Supplementary information

Supplementary Information.

## Data Availability

All data supporting the findings of this study are found in the article (and its Supplementary Information file).
