# Supplementary material for: Functional characterization of three Azotobacter chroococcum alginate-modifying enzymes related to the Azotobacter vinelandii AlgE mannuronan C-5-epimerase family
Source: Sci Rep. 2020 Jul 27;10:12470. doi: 10.1038/s41598-020-68789-3 (PMC7385640; doi:10.1038/s41598-020-68789-3)
Supplement: Supplementary file 1 — Supplementary Information. [file 41598_2020_68789_MOESM1_ESM.pdf]

**SUPPLEMENTARY INFORMATION**

# Functional characterization of three *Azotobacter chroococcum* alginate-modifying enzymes related to the *Azotobacter vinelandii* AlgE mannuronan C-5-epimerase family

Agnieszka Gawin (agnieszka.gawin@ntnu.no); Lisa Tietze (lisa.tietze@ntnu.no); Olav A. Aarstad (olav.a.aarstad@ntnu.no); Finn L. Aachmann (finn.l.aachmann@ntnu.no); Trygve Brautaset (trygve.brautaset@ntnu.no); Helga Ertesvåg(helga.ertesvag@ntnu.no)*

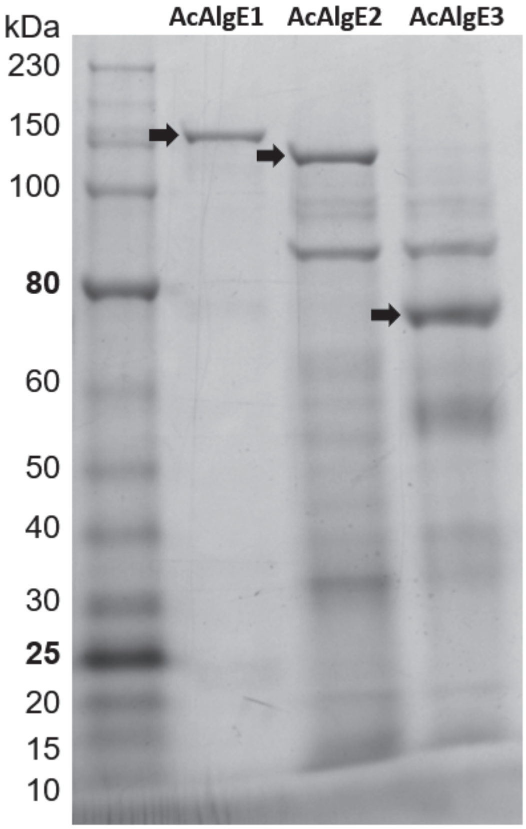


**Figure S1** Analysis of FPLC-purified recombinant AcAlgE1, AcAlgE2, and AcAlgE3 on an 4-12% polyacrylamide-SDS gel. The arrows indicate positions of the relevant proteins. The molecular masses deduced from the amino acid sequences are as follows: 104 kDa (AcAlgE1), 91 kDa (AcAlgE2), 52 kDa (AcAlgE3). The indicated bands correspond to a higher molecular mass than expected and this was also previously reported for other AlgE-type mannuronan C-5-epimerases (1).

1. Høidal, H. K., Ertesvåg, H., Skjåk-Braek, G., Stokke, B.T., Valla, S. The recombinant *Azotobacter vinelandii* mannuronan C-5-epimerase AlgE4 epimerizes alginate by a nonrandom attack mechanism. *J Biol Chem*. 1999;274(18):12316-22.
